# Intercropping system modulated soil–microbe interactions that enhanced the growth and quality of flue-cured tobacco by improving rhizospheric soil nutrients, microbial structure, and enzymatic activities

**DOI:** 10.3389/fpls.2023.1233464

**Published:** 2023-10-24

**Authors:** Muqiu Zhou, Chenglin Sun, Bin Dai, Yi He, Jun Zhong

**Affiliations:** ^1^ College of Agriculture, Hunan Agricultural University, Changsha, Hunan, China; ^2^ Technology center, Bijie Branch of Guizhou Tobacco Company, Bijie, Guizhou, China

**Keywords:** flue-cured tobacco, intercropping, rhizospheric soil, soil nutrients, enzymatic activity, microbial community structure

## Abstract

As the promotive/complementary mechanism of the microbe–soil–tobacco (*Nicotiana tabacum* L.) interaction remains unclear and the contribution of this triple interaction to tobacco growth is not predictable, the effects of intercropping on soil nutrients, enzymatic activity, microbial community composition, plant growth, and plant quality were studied, and the regulatory mechanism of intercropping on plant productivity and soil microenvironment (fertility and microorganisms) were evaluated. The results showed that the soil organic matter (OM), available nitrogen (AN), available phosphorus (AP), available potassium (AK), the urease activity (UE) and sucrase activity (SC), the diversity, abundance, and total and unique operational taxonomic units (OTUs) of bacteria and fungi as well as plant biomass in T1 (intercropping onion), T2 (intercropping endive), and T3 (intercropping lettuce) treatments were significantly higher than those of the controls (monocropping tobacco). Although the dominant bacteria and fungi at the phylum level were the same for each treatment, LEfSe analysis showed that significant differences in community structure composition and the distribution proportion of each dominant community were different. Proteobacteria, Acidobacteria, and Firmicutes of bacteria and Ascomycota and Basidiomycetes of fungi in T1, T2, and T3 treatments were higher than those of the controls. Redundancy analysis (RDA) suggested a close relation between soil characteristic parameters and microbial taxa. The correlation analysis between the soil characteristic parameters and the plant showed that the plant biomass was closely related to soil characteristic parameters. In conclusion, the flue-cured tobacco intercropping not only increased plant biomass and improved chemical quality but also significantly increased rhizospheric soil nutrient and enzymatic activities, optimizing the microbial community composition and diversity of rhizosphere soil. The current study highlighted the importance of microbe–soil–tobacco interactions in maintaining plant productivity and provided the potential fertilization practices in flue-cured tobacco production to maintain ecological sustainability.

## 1 Introduction

The growing demands for staple food and the limited arable land have resulted in flue-cured tobacco production with high cropping intensity and long monocropping periods (Chen et al., 2016; Fu et al., 2018; Li et al., 2022), but long-term monocropping may adversely affect the diversity and abundance of microbial community in the soil, thereby reducing soil quality, hindering the growth and development of tobacco plants, and ultimately affecting tobacco yield and quality (Wang, 2016; Gong et al., 2018; Ma et al., 2021).

However, intercropping, which simultaneously grows multiple crop species in a single field, has been widely practiced due to its economic, ecological, and environmental benefits (Martin-Guay et al., 2018; Dowling et al., 2021). Many researchers have shown that intercropping, in addition to affecting crop yield, may also cause functional and architectural alterations in the soil microbiota (Duchene et al., 2017; Yu et al., 2018). In the intercropping systems, the soil, microbes, and plants may interact with each other in various ways; e.g., microbes might influence soil nutrient turnover by decomposing soil organic matter (OM), which in turn influences soil enzymatic properties and secretion (Lauber et al., 2008; Peng et al., 2015). Soil enzymes facilitate the decomposition of soil microbes and plant debris, providing plants with nutrients to survive (Veres et al., 2015).

Different intercropping patterns may differentially affect soil physicochemical properties and microbial characteristics. Although previous studies have reported the tobacco–corn and tobacco–wheat (Zhou et al., 2015) intercropping systems, the effects of intercropping onion, endive, and lettuce with tobacco on soil nutrients, enzymatic activities, microbial community structure, and tobacco yield have been rarely reported. Therefore, we used high-throughput sequencing to investigate the impact of tobacco monocropping and intercropping systems on soil physio-biochemical and biological properties in Guizhou Province, which in turn affected tobacco productivity. The main objectives of the present study were as follows: 1) investigating the effects of tobacco intercropping with various other crops on the soil physio-biochemical properties, soil enzymatic activities, and tobacco yield; 2) comparing the differences in the microbial diversity and soil microflora composition between tobacco monocropping and intercropping; 3) determining the relationships among soil microbes, soil enzymatic activities, and physio-biochemical properties as well as flue-cured tobacco plants.

## 2 Materials and methods

### 2.1 Experimental design and sampling

The experiment was carried out in Weining County, Bijie City, Guizhou Province, China (103°80′E, 27°20′N, altitude 1,100 m) from April to October 2022, with the climate belonging to a humid subtropical monsoon and an average yearly temperature of 15.5°C, precipitation of 909 mm, and 1,812 hours of photoperiod. The prevailing soil composition in this geographical region primarily consists of clayey soil. The field has been continuously planted with tobacco for 10 years. The tested variety of flue-cured tobacco was the main local variety Yunyan 87, with a planting density of 16,500 plants/hectare at a row spacing of 1.2 m × 0.5 m.

Three treatments were used to intercrop the flue-cured tobacco: scallions (T1), endive (T2), and rapeseed (T3). A non-intercropped monoculture tobacco field was used as the control (CK). The total experimental area was 66.7 m^2^. After transplanting tobacco seedlings in April 2022, the intercropping plants were respectively on both sides of the flue-cured tobacco, with 82,500 plants/hectare, 33,000 plants/hectare, and 16,500 plants/hectare planted for scallions, endive, and rapeseed, respectively.

Rhizospheric soil samples were collected in June by scooping the surface soil around the tobacco plants to a depth of 3 cm, followed by digging with a shovel approximately 25 cm away from the stem of the tobacco plant. After the rhizosphere of the tobacco plant was exposed, the soil was taken as a sample. The whole process was replicated three times. The collected soil samples were temporarily stored in a polyethylene box with an ice pack and then completely homogenized through a soil screen of 2 mm. Each soil sample was equally divided into two parts. The samples for biochemical analysis were air-dried for 1 week and kept at −20°C. Samples for microbial community analysis were kept at −80°C (Zhao et al., 2019).

Biomass analysis was conducted three times during the entire growth period of tobacco plants. The destructive sampling was carried out at 30 days, 60 days, and 90 days after transplanting. Three representative tobacco plants were selected from each treatment with the roots, stems, and leaves dried for biomass measurement. After tobacco leaves were roasted, the contents of water-soluble sugar (including total sugar and reducing sugar), total alkali, total nitrogen, and potassium were determined with the tobacco industrial standards YC/T 159-2019, YC/T 160-2002, YC/T 33-1996, and YC/T 217-2007, respectively.

### 2.2 Soil properties and enzymatic activity

The available nitrogen (AN), available phosphorus (AP), available potassium (AK), and OM contents were measured following the instructions of the Kjeldahl method, the molybdenum antimony anti-chromogenic extraction method, inductively coupled plasma (ICP) spectrometer method, and the K2Cr2O7-H2SO4 oxidation approach (Zhao et al., 2020), respectively. Urease (UE), sucrase (SC), and peroxidase (POD) activities were measured using the phenol sodium hypochlorite colorimetric method, the 3,5-dinitrosalicylic acid colorimetric method, and spectrophotometry method (Jia, 2016), respectively.

### 2.3 Soil DNA extraction, PCR amplification, and sequencing

Total soil DNA was extracted from a 3-g soil sample using a Power Soil DNA Kit (MOBIO Inc., Carlsbad, CA, USA). PCR was performed to amplify the V3–V4 region of the bacterial 16S rRNA gene using primer pair 338F 5′-ACTCCTACGGGAGGCAGCA-3′ and 806R 5′-GGACTACHVGGGTWTCTAAT-3′. As for the fungal community, the ITS1 region of ITS gene was amplified using primer pair ITS1 5′-GGAAGTAAAAGTCGTAACAAGG-3′ and ITS2 5′-GCTGCGTTCTTCATCGATGC-3′. Finally, paired-end sequencing of the bacteria and fungi was performed on an Illumina MiSeq sequencer at Novogene Co., Ltd. (Beijing, China) (Wang, 2016).

### 2.4 Statistical analysis

The relative abundance of the rhizosphere soil microorganism community was analyzed by one-way ANOVA using SPSS 16.0 software. Uparse software was used to cluster Effective Tags into operational taxonomic units (OTUs) with a threshold value of 97% similarity, and the sequences with the largest number of OTUs were selected as the representative sequences of the OTUs for species annotation (citation). QIIME software was used to calculate the Alpha diversity index (citation). R language tool was used to make the composition of the community (citation). Circos software was used to analyze the composition proportion of the dominant community and its distribution proportion in samples (xxxx). LEfSe software was used to analyze soil communities with significant differences, and principal coordinate analysis (PCoA) in R was used to analyze the difference in community structure (xxxx). Tax4Fun and FUNGuild methods were used to compare the existing 16S rRNA and ITS gene sequencing data with the SILVA database used to compare the abundance differences of functional genes in biological metabolic pathways (xxxx). The “vegan” redundancy analysis R software (RDA) and Mantel test were used to analyze the relationship between soil nutrients, enzymatic activities, and microbial community (Tang et al., 2020).

## 3 Results

### 3.1 Effects of intercropping on soil nutrients and enzymatic activities in the rhizospheric soil of flue-cured tobacco

The OM, AN, AP, and AK contents in the soil of the intercropping treatment groups of flue-cured tobacco were significantly higher than those in CK (**Table 1**), in which T1 treatment reached the highest. Compared to the controls, T1, T2, and T3 increased OM content by 44.87%, 34.55%, and 40.32%, respectively; the AN contents were increased by 36.59%, 26.85%, and 33.12%, respectively; the AP contents were increased by 48.23%, 28.17%, and 36.84%, respectively; the AK contents were increased by 39.61%, 21.08%, and 30.59%, respectively. The enzymatic activity of the soil under intercropping treatments (T1, T2, and T3) decreased by 14.49%, 5.15%, and 7.30% in terms of POD contents, respectively. The UE contents were increased by 46.42%, 11.81%, and 34.48%, respectively, and the SC contents were increased by 40.92%, 17.94%, and 31.67%, respectively.

**Table 1:** Soil nutrients and enzymatic activity of the soil.

Treatment	Organic matter (g/kg)	Available nitrogen (mg/kg)	Available phosphorus (mg/kg)	Available potassium (mg/kg)	Peroxidase activity (U/g)	Urease activity (U/g)	Sucrase activity (U/g)
CK	14.73 ± 0.38c	62.49 ± 0.15b	127.39 ± 0.45c	56.92 ± 0.46b	38.64 ± 0.39c	65.86 ± 0.42c	188.31 ± 0.57b
T1	21.34 ± 0.22a	85.36 ± 0.26a	188.84 ± 0.37a	79.47 ± 0.38a	33.04 ± 0.31a	96.43 ± 0.28a	265.37 ± 0.46a
T2	19.82 ± 0.36b	79.27 ± 0.45ab	163.27 ± 0.33b	68.92 ± 0.43ab	36.65 ± 0.18b	73.64 ± 0.41b	222.09 ± 0.51ab
T3	20.67 ± 0.55ab	83.19 ± 0.39a	174.33 ± 0.28ab	74.33 ± 0.22a	35.82 ± 0.27a	88.57 ± 0.37ab	247.95 ± 0.37a

Different lowercase letters indicate significant differences (p < 0.05).

### 3.2 Effects of intercropping on microbial community diversity and structure in the rhizosphere soil of flue-cured tobacco

#### 3.2.1 Microbial community diversity

**Figure 1:**
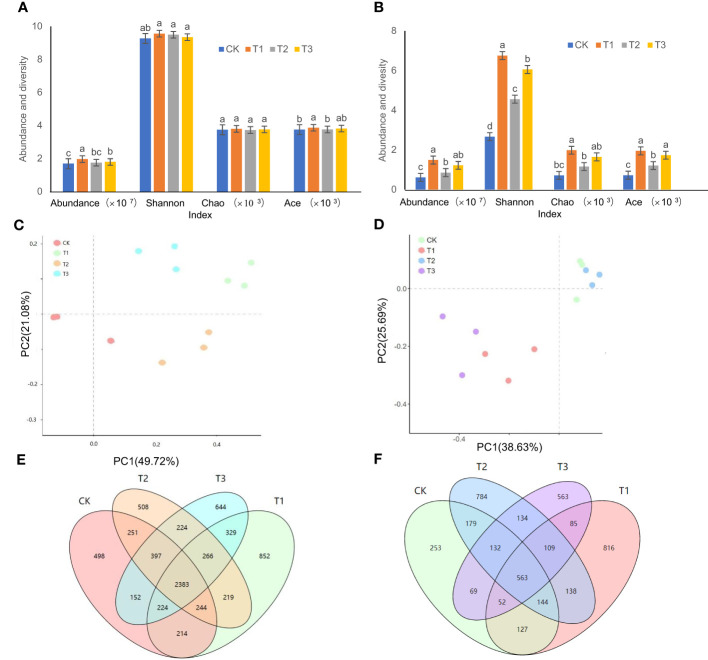
Microbial abundance and diversity in the rhizosphere soil of flue-cured tobacco under different intercropping treatments. **(A)** Abundance and diversity of bacteria; **(B)** Abundance and diversity of fungi; **(C)** PCoA analysis of bacteria; **(D)** PCoA analysis of fungi; **(E)** OTU analysis of bacteria; **(F)** OTU analysis of fungi. Different lowercase letters above the column indicate significant differences (p<0.05), the same as below.

The order of bacterial abundance was ranked as T1>T3>T2>CK, and the bacterial abundance in T1 was significantly higher than that in T2 and T3 treatments (**Figure 1A**) . Compared to the controls, the bacterial abundance in the T1, T2, and T3 treatments increased by 16.52%, 12.10%, and 6.28%, respectively. Among the Shannon, Ace, and Chao diversity indices of the T1, T2, and T3 treatments, only Ace indices between T1 and T2 treatments showed significant differences (**Figure 1A**). The PCoA showed (**Figure 1C**) that there was an obvious separation between the four treatments, with the X and Y axes explaining 49.72% and 21.08% of the overall variation of the bacterial population, respectively. The OTU analysis showed that the unique bacterial OTUs of CK, T1, T2, and T3 were 498, 852, 508, and 644, respectively (**Figure 1E**).

The changes in fungal abundance as well as the three Alpha diversity indices of Shannon, Ace, and Chao were ordered as T1>T3>T2>CK (**Figure 1B**). The differences between the T2 and the control were not significant, but both of them were significantly lower than the T1 and T3 treatments (**Figure 1B**). PCoA results indicated that the X and Y axes explained 38.63% and 25.69% of the overall variation of the fungal community, respectively (**Figure 1D**). The unique OTUs of the tobacco fungi in the rhizospheric soil were ranked as T1 (816)>T2 (784)>T3 (563)>CK (253) (**Figure 1F**).

#### 3.2.2 Microbial community structure

**Figure 2:**
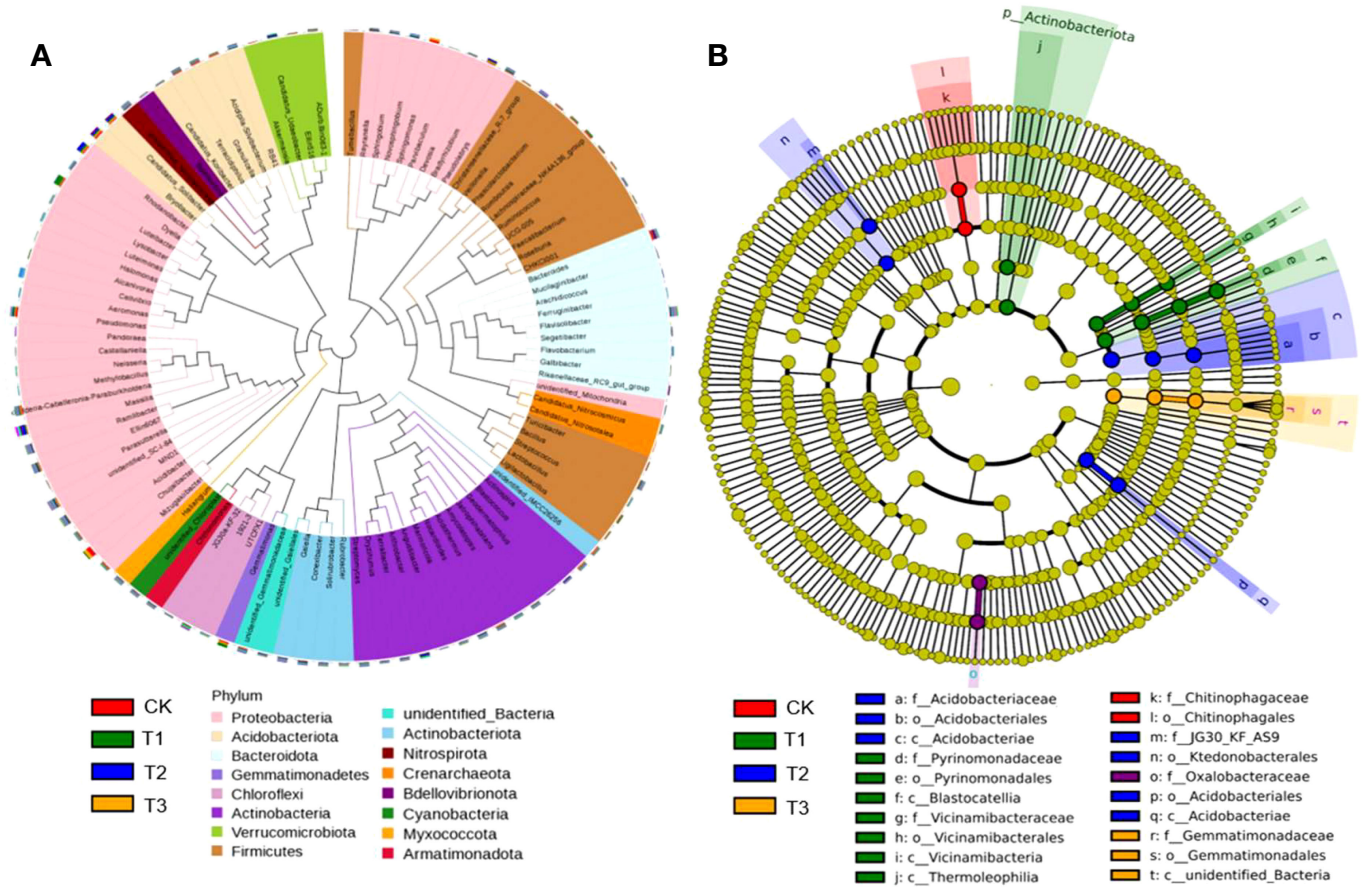
Bacterial dominant communities in the rhizosphere soil of flue-cured tobacco under different intercropping treatments. **(A)** Evolutionary map of phylum level of bacteria; **(B)** LEfSe analysis of differences in bacterial community structure composition.

Although the bacteria with relative abundance>3% at the phylum level were the same, Proteobacteria, Acidobacteria, Bacteroidota, Gemmatimonadetes, Firmicutes, and Actinobacteriota were the main bacterial communities. These were present in much higher quantities than other dominant bacterial groups and accounted for approximately 86.73% of the total bacterial groups (**Figure 2A**). However, the community structure composition from the phylum level to the genus level among different treatments was shown from the LEfSe analysis. Among them, nine groups in T1, seven groups in T2, three groups in T3, and two groups in CK were identified as differential bacterial communities (**Figure 2B**).

**Figure 3:**
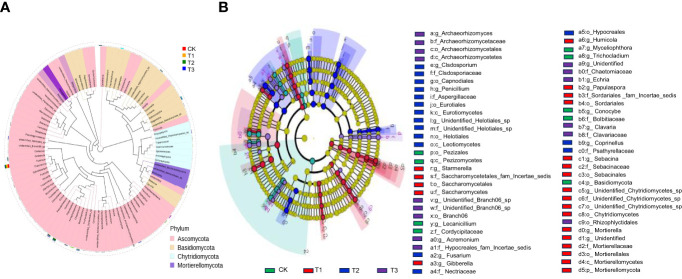
Fungal dominant communities in the rhizosphere soil of flue-cured tobacco under different intercropping treatments.**(A)** Evolutionary map of phylum level of fungi; **(B)** LEfSe analysis of differences in fungi community structure composition

The top 100 fungi in each treatment group at the genus level belonged to six phyla, namely, Ascomycota, Basidiomycota, Chytridiomycota, Mortierellomycota, Mucormycota, and Glomeromycota, which accounted for 78.11%–95.32% of the total fungi (**Figure 3A**). However, the community structure composition among different treatments identified 22 groups in the T1 treatment, 16 groups in the T2 treatment, 15 groups in the T3 treatment, and 9 groups in the control as differential fungal communities (**Figure 3B**).

#### 3.2.3 Effects of intercropping on the distribution of dominant microorganism communities

**Figure 4:**
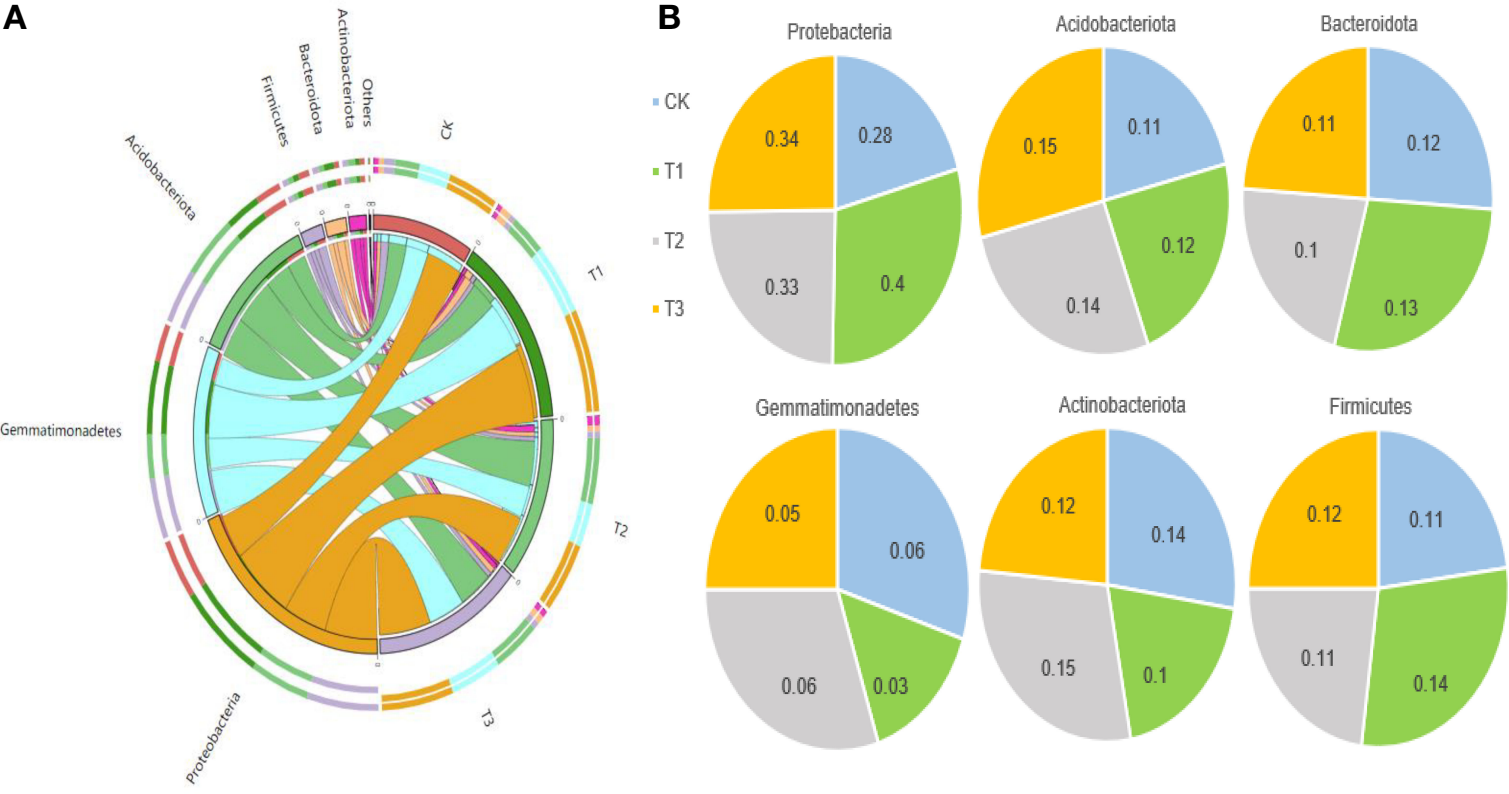
The distributions of bacterial dominant communities in the rhizosphere soil of flue-cured tobacco under different intercropping treatments. **(A)** Circos analysis of dominant fungal composition; **(B)** Distribution ratio of dominant fungi of phyla level in different treatments.

**Figure 5:**
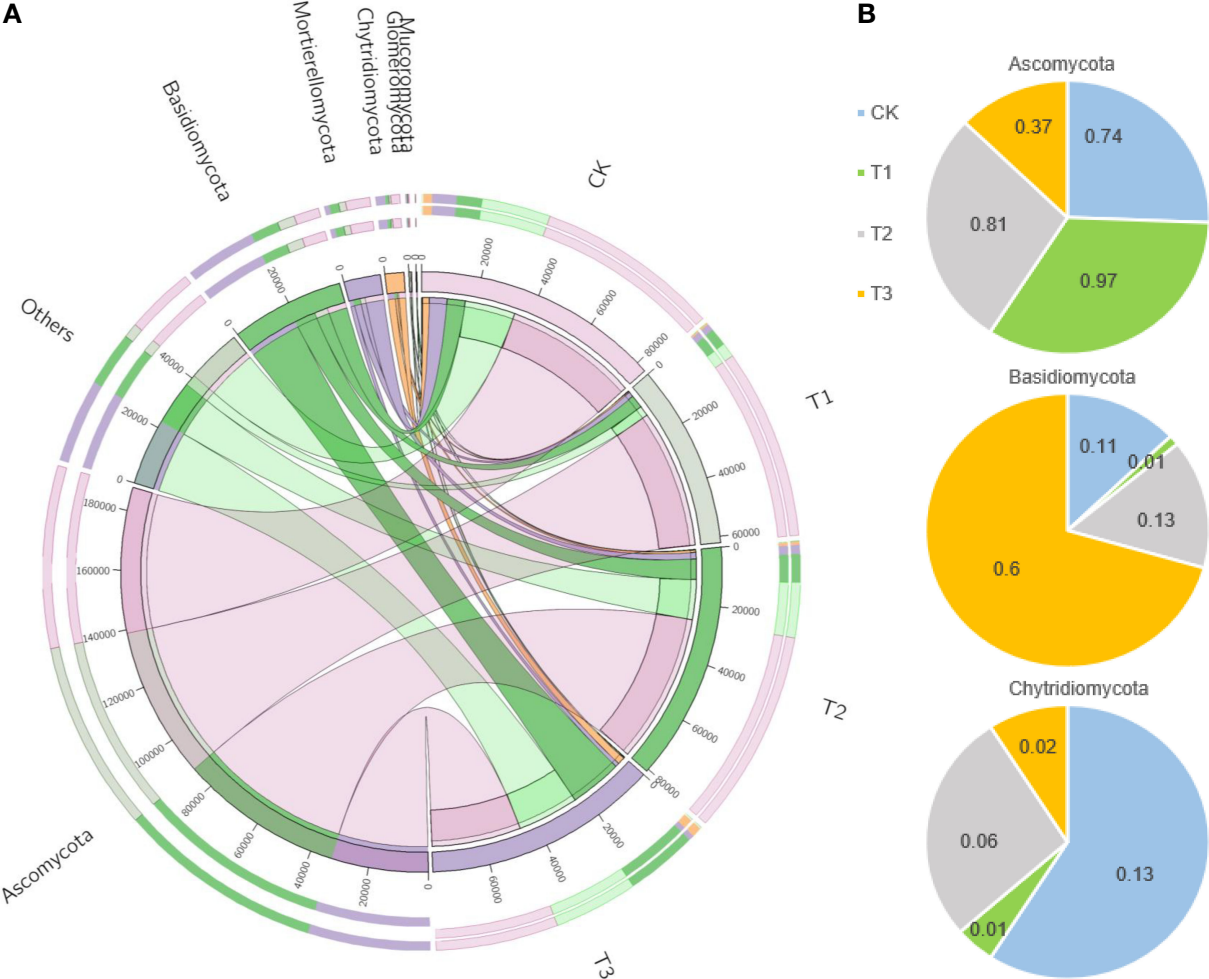
The distributions of fungal dominant communities in the rhizosphere soil of flue-cured tobacco under different intercropping treatments. **(A)** Circos analysis of dominant bacterial composition; **(B)** Distribution ratio of dominant bacteria of phyla level in different treatments.

The Circos analysis showed the distributions of dominant bacteria3and fungi in different treatments (**Figures 4A**, **5A**). Based on the phylum level of bacteria, the proportions were ranked as T1 (40%)>T3 (34%)>T2 (33%)>CK (28%) for Proteobacteria, T3 (15%)>T2 (14%)>T1 (12%)>CK (11%) for Acidobacteria, T1 (13%)>CK (12%)>T3 (11%)>T2 (10%) for Bacteroides, T2=CK (6%)>T3 (5%)>T1 (3%) for Gemmatimonadetes, T2 (15%)>CK (14%)>T3 (12%)>T1 (10%) for Actinobacteria, and T1 (14%)>T3 (12%)>T2=CK (11%) for Firmicutes (**Figure 4B**).

Based on the phylum level of fungi with the proportion larger than 1%, Ascomycota was distributed as T1 (97.22%)>T2 (80.89%)>CK (74.21%)>T3 (37.21%), Basidiomycetes as T3 (60.51%)>T2 (12.81%)>CK (11.77%)>T1 (1.26%), and *Mortierella* as CK (12.86%)>T2 (5.91%)>T3 (2.23%)>T1 (1.41%) (**Figure 5B**).

### 3.3 Effects of intercropping on growth and quality of flue-cured tobacco plants

**Figure 6:**
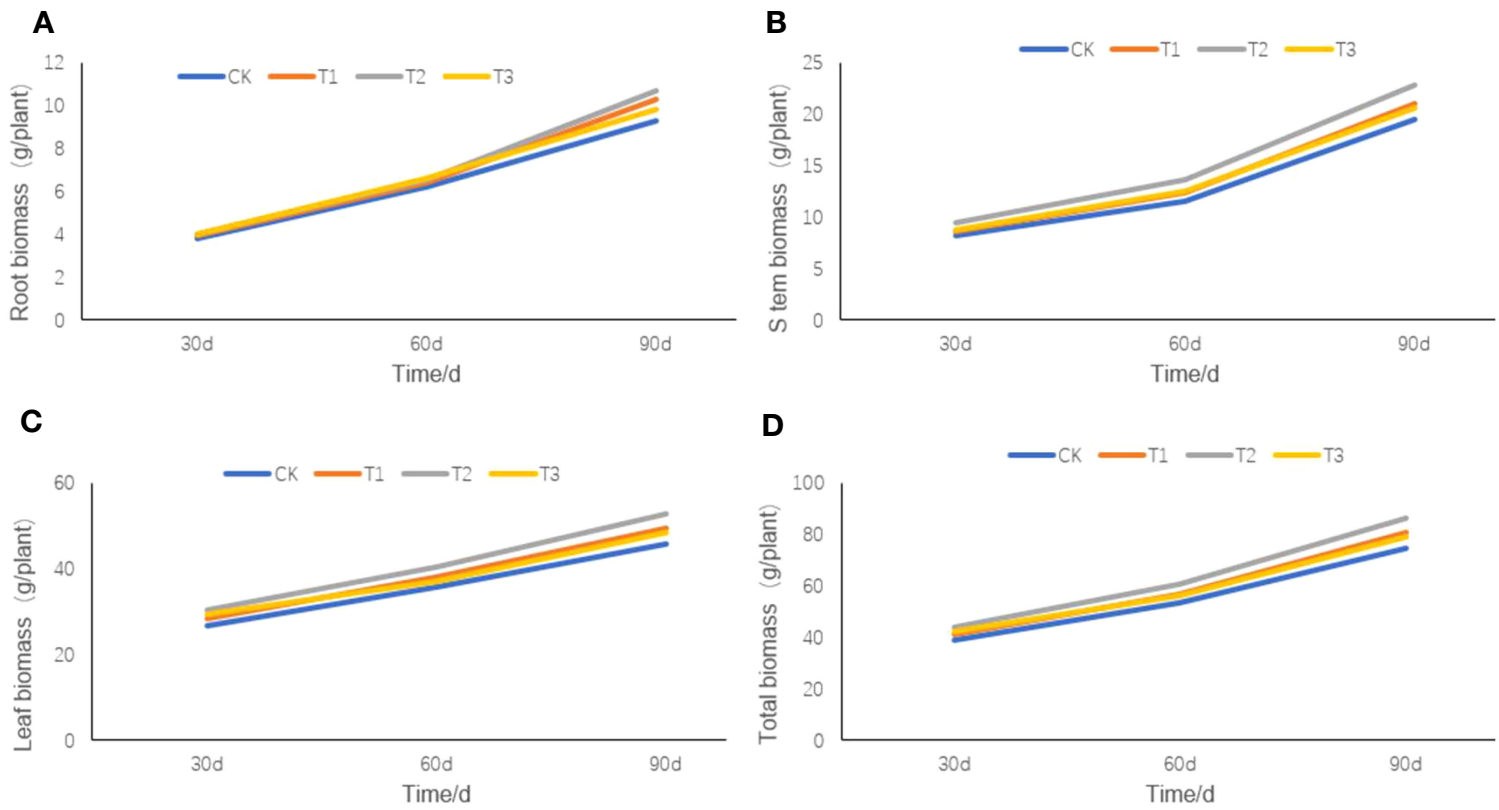
The root, stem, and leaf biomass of flue-cured tobacco under different intercropping treatments. **(A)** the root biomass of tobacco plant; **(B)** the stem biomass of tobacco plant; **(C)** the leaf biomass of tobacco plant; **(D)** the total biomass of tobacco plant.

Except for the biomass of tobacco root (**Figure 6A**), stem (**Figure 6B**), leaf (**Figure 6C**), or total biomass (**Figure 6D**) on the 30th day after transplanting, little differences were observed among the four treatments. However, on the 60th day after transplanting, there were slight differences in the root, stem, leaf, and total biomass of the four treatments, among which the T2 treatment was the largest and the CK treatment was the smallest. On the 90th day after transplanting, significant differences appeared in the total biomass of the roots, stems, and leaves among the four treatments, but they were still the largest in the T2 treatment and the smallest in the CK treatment.

When compared with the control, the tobacco leaves of the three intercropping systems were closer to the standard of high-quality tobacco leaves (**Table 2**).

**Table 2:** Routine chemical composition of different intercropping treatments.

Treatment	Total sugar%	Reducing sugar%	Total nitrogen%	Nicotine%	Potassium%	Chlorine%
Contrast	23.67 ± 1.51a	23.12 ± 4.92a	3.03 ± 1.27a	2.80 ± 0.97a	1.65 ± 0.44b	0.31 ± 0.11a
T1	21.33 ± 0.74b	19.42 ± 3.17b	2.75 ± 1.19ab	2.60 ± 1.04b	1.76 ± 0.28ab	0.22 ± 0.06b
T2	20.86 ± 1.03bc	18.38 ± 2.46b	2.59 ± 2.22b	2.46 ± 1.37bc	1.89 ± 0.32a	0.17 ± 0.14b
T3	22.59 ± 0.95ab	20.27 ± 4.28ab	2.86 ± 1.68ab	2.68 ± 1.46ab	1.63 ± 0.47b	0.21 ± 0.25b
High-quality tobacco	18–24	16–22	1.–3.5	1.5–3.5	>2	<1

### 3.4 Correlation analysis

#### 3.4.1 Redundancy analysis between dominant microbial communities and soil nutrients

**Figure 7:**
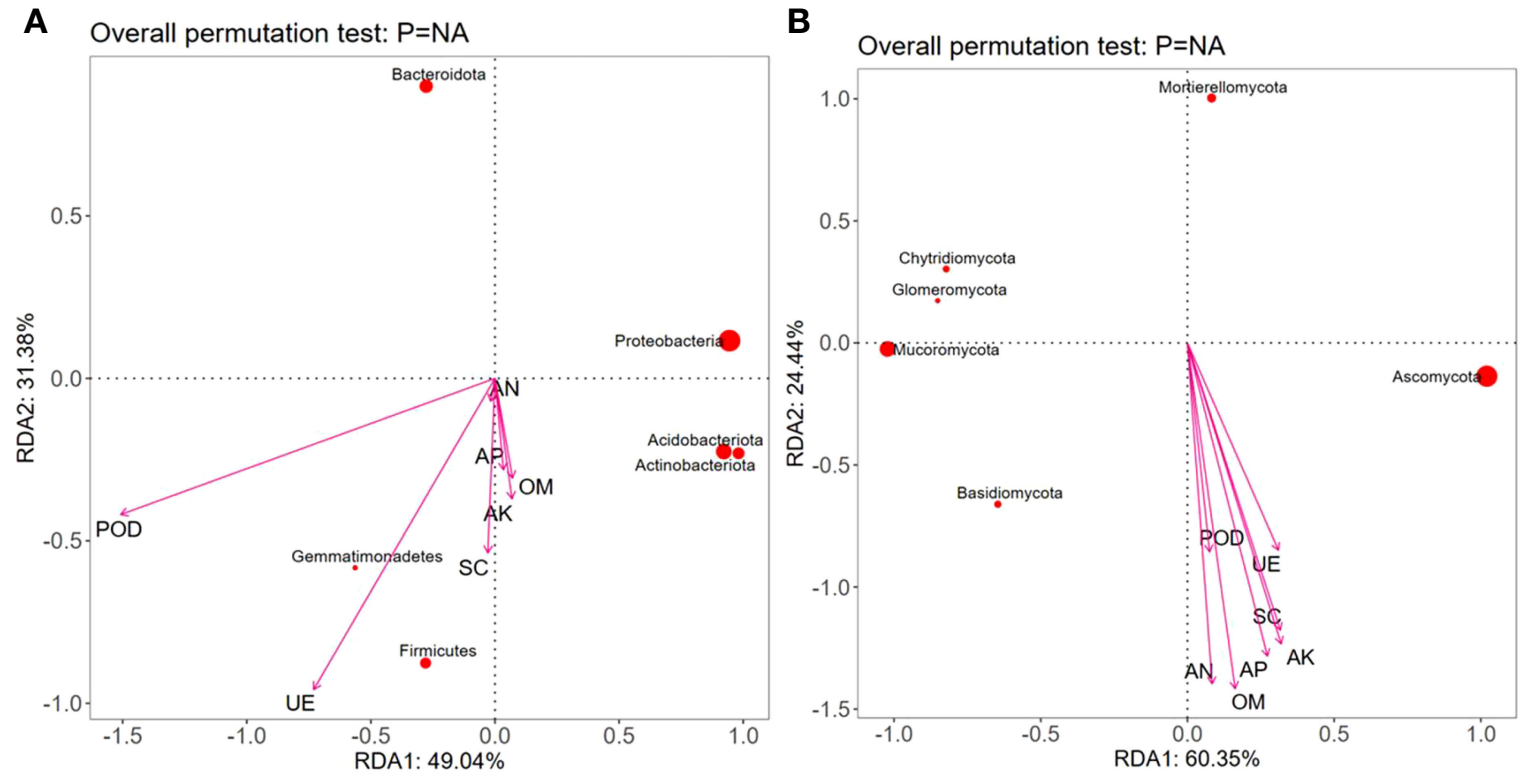
Redundancy analysis (RDA) between dominant microbial communities, nutrients, and enzymatic activity in the rhizospheric soil under different intercropping treatments. Note: OM, AN, AP, AK, UE, SC, and POD represent respectively organic matter, available nitrogen, available phosphorus, available potassium, urease, sucrase, and peroxidase. **(A)** Redundancy analysis of soil nutrients and bacterial communities; **(B)** Redundancy analysis of soil nutrients and fungal communities.

RDA showed that the contents of OM, AN, and AP were positively correlated with Proteobacteria, Acidobacteria, Gemmatimonadetes, Actinobacteriota, and Firmicutes but negatively correlated with Bacteroidota; the contents of AK, UE, and SC were positively correlated with Acidobacteria, Gemmatimonadetes, Actinobacteriota, and Firmicutes but negatively correlated with Proteobacteria and Bacteroidota. The POD activity was positively correlated with Gemmatimonadetes and Firmicutes but negatively correlated with Proteobacteria, Acidobacteria, Actinobacteriota, and Bacteroidobacteria (**Figure 7A**). Moreover, various soil nutrient contents (OM, AN, AP, and AK) and enzymatic activities (POD, UE, and SC) were positively correlated with Ascomycota and Basidiomycota but negatively correlated with Chytridiomycota, Mortierellomycota, Mucoromycota, and Glomeromycota (**Figure 7B**).

#### 3.4.2 Correlation analysis between soil properties and flue-cured tobacco plants

**Figure 8:**
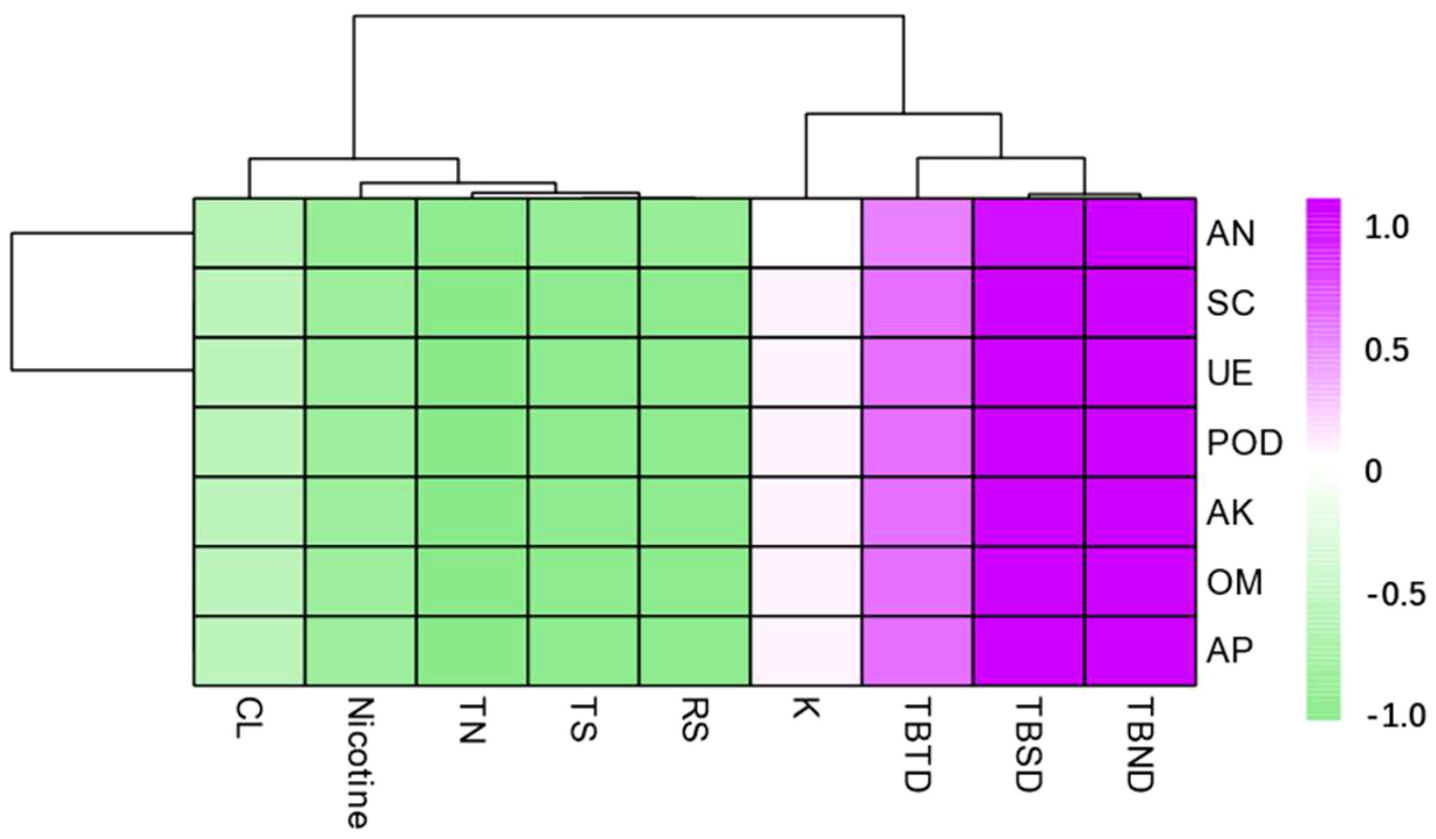
Correlation analysis between dominant microbial communities in the rhizosphere soil and total biomass, routine chemical composition under different intercropping treatments. TBTD, TBSD, TBND, TS, RS, and TN represent respectively total biomass of 30 days, total biomass of 60 days, total biomass of 90 days, total sugar, reducing sugar, and total nitrogen.

According to the correlation analysis, the total biomass was positively correlated with all the soil nutrients and enzymatic activity. The total biomass after 60 days and 90 days of transplanting reached a significant level at p < 0.01, while the total biomass after 30 days of tobacco plant transplanting reached a significant level at p < 0.05. The contents of RS, TS, TN, and nicotine were significantly negatively correlated with all the soil nutrients and enzymatic activities (**Figure 8**).

## 4 Discussion

### 4.1 Soil nutrients and enzymatic activities in the intercropped tobacco fields

Intercropping has become increasingly popular over the past decade to maintain soil biodiversity and improve nutrient content (Dai et al., 2019). One of the biggest advantages of intercropping is to make use of the interaction among different plants to improve soil physio-biochemical properties and enzymatic activities as well as to promote crop growth and development (Gong et al., 2019). Previous studies have demonstrated that intercropping peanuts with maize changed the abundance of nitrogen-fixing microbes in the rhizosphere (Chen et al., 2018). Cassava–peanut intercropping enriched Actinomycetes in the soil rhizosphere and boosted the absorption of soil-available nutrients, thus increasing the peanut yield (Chen et al., 2020). The present study also verified that the nutrients of all flue-cured tobacco intercropping treatments were significantly higher than those of the controls (**Table 1**), suggesting that intercropping could contribute to elevating the levels of soil nutrients.

Soil enzymatic activity is a vital parameter indicating how organic matter is degraded and the nutrients are cycled in the soil (Nannipieri et al., 2012; Hussain et al., 2021). The soil enzymatic activity can be represented by the physio-biochemical traits and microbial communities (Gu et al., 2009). For example, sucrase hydrolyzes sucrose, which reflects the convertibility of the soil organic carbon, while urease impacts the metabolism of soil nitrogen through urea hydrolyzation (Cantarella et al., 2018). In this study, the intercropping soil systems (T1, T2, and T3) presented the highest UE and SC activities compared to the CK (**Table 1**). Likewise, Zhou et al. (2011) also reported elevation in the soil urease activity in the cucumber–garlic/onion intercropping system compared to the monocropping system. Peroxidase activity is considered a crucial predictor of the dynamics of soil organic matter (Tian and Shi, 2014).

### 4.2 Effects of intercropping on the soil structure of microbial communities

The intercropping systems led to alterations in the soil physio-biochemical parameters and enzymatic activities, prompting the enrichment of a particular subset of functional bacteria and fungi, which was manifested as elevated diversities of both microbial types in the intercropped soil compared to the tea monoculture (Zhang et al., 2019; Bai et al., 2022; Jing et al., 2022). In agreement with this, an increase in the abundance and diversity of microbial communities in the rhizospheric soil of flue-tobacco plants was also observed under the three intercropping treatments (**Figures 1A, B**). This may be due to the fact that the plant root distribution in the top layer of the soil was expanded by intercropping. Thus, the permeability of the soil increased and the circulation of nutrient elements became more efficient, which provided a better microenvironment for the growth and propagation of microorganisms.

Intercropping may also influence soil microbiota composition differences (Bainard et al., 2012a; Floc’h et al., 2020) In this study, the PCoA of soil bacterial and fungal communities revealed a separation between the four treatments, indicating that the microbiota composition differed between the monoculture and intercropped plants (**Figures 1C, D**). The dominant microbial community in the rhizospheric soil of tobacco plants under four treatments was the same (**Figures 4A**, **5A**), which is in agreement with previous studies (Zhang et al., 2017; De Vries et al., 2020), but the dominant community composition and their ratio were different (**Figures 2**, **3**, **4B**, **5B**), which roughly corresponded to previous studies investigating agricultural soils (Li et al., 2011; Bai et al., 2020). Previous reports indicated that many members of Proteobacteria and Bacteroidetes of the soil bacteria community were closely associated with the carbon (C) and nitrogen (N) cycles (Leff et al., 2015; Pardon et al., 2017), while Ascomycota of the soil fungal community can rapidly metabolize organic substrates of rhizodeposition in rhizosphere soil (Bastida et al., 2013). As a result, the intercropping systems might increase soil nutrient accumulation and nutrient utilization efficiency by promoting microbial growth that is closely associated with N fixation or other C–N processes. In this study, the abundance of Proteobacteria of bacteria and Ascomycota of fungi in the intercropping systems was significantly higher than the monocropping ones (**Figures 4**, **5**), indicating the improved soil nutrient conditions after intercropping.

### 4.3 Effects of intercropping on tobacco growth and quality

Positive interactions occur in complex symbiotic systems that enhance the growth of crops, and these interactions are beneficial for improving soil fertility and yield (Hauggaard et al., 2001; Qian et al., 2018). In this study, intercropping can substantially increase the biomass (**Figure 6**) and improve the quality of routine chemical composition (**Table 2**) of tobacco plants. The growth and quality improvement results of the tobacco plants are consistent with previous research on *Aconitum carmichaeli*–rice intercropping systems (Ren et al., 2018). These results revealed that belowground interaction in intercropping can induce changes in soil microbial community structure, such as the increase in Acidobacteria abundance of bacteria can better degrade plant residue polymers and enhance photosynthesis, thereby increasing soil nutrients and plant biomass; the increase of plant residue polymers also promoted the increase of Ascomycota abundance of fungi, and the fungi can rapidly metabolize organic substrates of rhizodeposition in rhizosphere soil (Bastida et al., 2013). Therefore, we concluded that the increased microbial abundance of the tobacco intercropping system could accelerate the soil nutrients cycle, which in turn may have been beneficial to maintaining high soil fertility, growth, and quality of plants.

### 4.4 Soil–microbe–plant interaction

The direct and positive relationships between microbial diversity and soil nutrients in this study (**Figure 1**) coincide with the documented finding that soils with high microbial community diversity typically have increased nutrient availability (Zhang et al., 2021). This demonstrated that a diverse microbial community provides more nutrients for plants to absorb and less competition from microorganisms. Increased microbial diversity may promote the accumulation of available N and OM in the soil, all of which could increase overall plant productivity (Banerjee et al., 2018; Delgado-Baquerizo et al., 2020). Moreover, three intercropping treatments increased plant biomass (**Figure 6**) and improved the chemical composition of tobacco leaves (**Table 2**), which strongly suggests the potential role of intercropping on plant performance. Higher tobacco biomass and soil fertility of intercropping may depend on the regulating ability of belowground interactions since soils with high diversity also have higher plant productivity (Lan et al., 2023) and decomposition ability in organic matters (Liu et al., 2020b). In summary, intercropping could increase the abundance of soil microbes, which can promote the release of soil enzymes to catalyze various biochemical reactions such as mineralization of organic matter, synthesis of humus substances, and release of growth active substances, thus improving the overall growing environment and ultimately improving the biomass and quality of the flue-cured tobacco.

## 5 Conclusion

Our study suggested that intercropping flue-cured tobacco with other plants could increase the contents of soil AN, AP, AK, and OM and greatly improve soil nutrient status, thereby facilitating the improvement of the growth and quality of the flue-cured tobacco plants. Meanwhile, intercropping also altered the microbial community structure in the soil rhizosphere, while the microbial structure was closely related to the soil characteristics. Overall, intercropping may directly or indirectly increase plant productivity by regulating soil fertility and microbial dynamics in the rhizosphere of flue-cured tobacco. Therefore, soil–microbe–plant interactions should be promoted in arable systems to improve sustainable crop productivity.

## Data availability statement

The data presented in the study are deposited in the BioSample database repository, accession number SAMN35711959, SAMN35711960, SAMN35711961, SAMN35711962.

## Author contributions

MZ and CS performed the statistical analysis, and the preliminary manuscript was composed by JZ. BD and YH contributed to the conception and design of the study. MZ and CS are the co-authors, and JZ and YH are the co-corresponding authors. All authors contributed to the article and approved the submitted version.

## References

[B1] Bai Y. C. Chang Y. Y. Hussain M. Lu B. Zhang J. P. Song X. B.. (2020). Soil chemical and microbiological properties are changed by long-term chemical fertilizers that limit ecosystem functioning. Microorganisms 8, 694. doi: 10.3390/microorganisms8050694 32397341 PMC7285516

[B2] Bai Y. C. Li B. X. Xu C. Y. Raza M. Wang Q. Wang Q. Z.. (2022). Intercropping walnut and tea: Effects on soil nutrients, enzyme activity, and microbial communities. Front. Microbiol. 13, 576–589. doi: 10.3389/fmicb.2022.852342 PMC8971985 35369467

[B3] Bainard L. D. Koch A. M. Gordon A. M. Klironomos J. N. (2012a). Growth response of crops to soil microbial communities from conventional monocropping and tree-based intercropping systems. Plant Soil 363, 345–356. doi: 10.1007/s11104-012-1321-5

[B4] Banerjee S. Schlaeppi K. van der Heijden M. G. A. (2018). Keystone taxa as drivers of microbiome structure and functioning. Nat. Rev. Microbiol. 16 (9), 567–576. doi: 10.1038/s41579-018-0024-1 29789680

[B5] Bastida F. Hernández T. Albaladejo J. García C. (2013). Phylogenetic and functional changes in the microbial community of long–term restored soils under semiarid climate. Soil Biol. Biochem. 65, 12–21. doi: 10.1016/j.soilbio.2013.04.022

[B6] Cantarella H. Otto R. Soares J. R. (2018). Agronomic efficiency of NBPT as a urease inhibitor: A review. J. Adv. Res. 13, 19–27. doi: 10.1016/j.jare.2018.05.008 30094079 PMC6077139

[B7] Chen J. Arafat Y. Wu L. K. Xiao Z. G. Li Q. S. Khan M. A.. (2018). Shifts in soil microbial community, soil enzymes and crop yield under peanut/maize intercropping with reduced nitrogen levels. Appl. Soil Ecol. 124, 327–334. doi: 10.1016/j.apsoil.2017.11.010

[B8] Chen Y. Bonkowski M. Shen Y. Griffiths B. S. Jiang Y. J. Wang X. Y.. (2020). Root ethylene mediates rhizosphere microbial community reconstruction when chemically detecting cyanide produced by neighbouring plants. Microbiome 8, 4. doi: 10.1186/s40168-019-0775-6 31954405 PMC6969408

[B9] Chen J. F. Sun H. Xia Y. Cai K. X. Liu H. Y. Zhao S. H.. (2016). Changes in soil enzyme activity and nutrient content in tobacco fields under different continuous cropping years. Henan Agric. Sci. 45 (10), 60–64. doi: 10.15933/j.cnki.1004-3268.2016.10.014

[B10] Dai J. Qiu W. Wang N. Wang T. Nakanishi H. Zuo Y. (2019). From Leguminosae/Gramineae intercropping systems to see benefits of intercropping on iron nutrition. Front. Plant Sci. 10. doi: 10.3389/fpls.2019.00605 PMC6527889 31139203

[B11] Delgado-Baquerizo M. Reich P. B. Trivedi C. Eldridge D. J. Abades S. Alfaro F. D.. (2020). Multiple elements of soil biodiversity drive ecosystem functions across biomes. Nat. Ecol. Evol. 4 (2), 210–220. doi: 10.1038/s41559-019-1084-y 32015427

[B12] De Vries F. T. Griffiths R. I. Knight C. G. Nicolitch O. Williams A. (2020). Harnessing rhizosphere microbiomes for drought-resilient crop production. Science 368 (6488), 270–274.doi:10.1126/science.aaz5192. doi: 10.1126/science.aaz5192 32299947

[B13] Dowling A. Sadras O. Roberts P. Doolette A. Zhou Y. Denton M. D. (2021). Legume-oilseed intercropping in mechanised broadacre agriculture – a review. Field Crop Res. 260, 107980. doi: 10.1016/j.fcr.2020.107980

[B14] Duchene O. Vian J. F. Celette F. (2017). Intercropping with legume for agroecological cropping systems: complementarity and facilitation processes and the importance of soil microorganisms. A review. Agric. Ecosyst. Environ. 240, 148–161. doi: 10.1016/j.agee.2017.02.019

[B15] Floc’h J. B. Hamel C. Harker K. Arnaud M. (2020). Fungal communities of the canola rhizosphere: keystone species and substantial between-year variation of the rhizosphere microbiome. Microbial Ecol. 80 (4), 762–777. doi: 10.1007/s00248-019-01475-8 31897569

[B16] Fu Z. Y. Zhang X. Y. Zhang X. F. Zhou H. J. Qin Y. H. Ma J.. (2018). Effects of continuous cropping of tobacco on soil carbon pool and post-curing tobacco leaf quality. J. Northwest A&F Univ. (Natural Sci. Edition) 46 (08), 16–22. doi: 10.13207/j.cnki.jnwafu.2018.08.003

[B17] Gong X. Liu C. Li J. Luo Y. Yang Q. Zhang W.. (2019). Responses of rhizosphere soil properties, enzyme activities, and microbial diversity to intercropping patterns on the Loess Plateau of China. Soil Tillage Res. 195, 1022–1033. doi: 10.1016/j.still.2019.104355

[B18] Gong Z. X. Ma X. H. Ren Z. G. Zhu J. F. Huang Y. J. Wang M. M.. (2018). Analysis of bacterial community in rhizosphere soil of continuously cropped tobacco using 16S rDNA-PCR-DGGE. China Agric. Sci. Technol. Tribune 20 (2), 39–47. doi: 10.13304/j.nykjdb.2017.0231

[B19] Gu Y. Wang P. Kong C. H. (2009). Urease, invertase, dehydrogenase, and polyphenoloxidase activities in paddy soil influenced by allelopathic rice variety. Eur. J. Soil Biol. 45 (5), 436–441. doi: 10.1016/j.ejsobi.2009.06.003

[B20] Hauggaard N. H. Ambus P. Jensen E. S. (2001). Interspecific competition, N use and interference with weeds in pea–barley intercropping. Field Crops Res. 70, 101–109. doi: 10.1016/S0378-4290(01)00126-5

[B21] Hussain S. Shafiq I. Skalicky M. Brestic M. Rastogi A. Mumtaz M.. (2021). Titanium application increases phosphorus uptake through changes in Auxin content and later root formation in soybean. Front. Plant Sci. 12. doi: 10.3389/fpls.2021.743618 PMC8631872 34858450

[B22] Jia L. Q. (2016). Effects of soil leachate from leguminous plants on soil microbial community and enzyme activity in continuous cropping of potatoes. [Master’s thesis (Gansu Agricultural University).

[B23] Jing Y. Z. Guo X. H. Wang X. L. Niu H. W. Han D. Xu Z. C. (2022). Effects of ginger intercropping on yield and quality of flue-cured tobacco, soil bacterial population, and physicochemical properties. Shandong Agric. Sci. 54 (1), 86–94. doi: 10.14083/j.issn.1001-4942.2022.01.014

[B24] Lan Y. Wang S. Zhang H. He Y. Jiang C. Ye S. (2023). Intercropping and nitrogen enhance eucalyptus productivity through the positive interaction between soil fertility factors and bacterial communities along with the maintenance of soil enzyme activities. Land Degrad. Dev. 4616, 1–15. doi: 10.1002/ldr.4616

[B25] Lauber C. L. Strickland M. S. Bradford M. A. Fierer N. (2008). The influence of soil properties on the structure of bacterial and fungal communities across land-use types. Soil Biol. Biochem. 40 (9), 2407–2415. doi: 10.1016/j.soilbio.2008.05.021

[B26] Leff J. W. Jones S. E. Prober S. M. Barberan A. Borer E. T. Firn J. L.. (2015). Consistent responses of soil microbial communities to elevated nutrient inputs in grasslands across the globe. Proc. Natl. Acad. Sci. U.S.A. 112, 10967–10972. doi: 10.1073/pnas.1508382112 26283343 PMC4568213

[B27] Li Y. Lee C. G. Watanabe T. Murase J. Asakawa S. Kimura M. (2011). Identification of microbial communities that assimilate substrate from root cap cells in an aerobic soil using a DNA-SIP approach. Soil Biol. Biochem. 43, 1928–1935. doi: 10.1016/j.soilbio.2011.05.016

[B28] Li Z. J. Zhu W. Q. Huang K. Ji X. W. Wang C. Zhao T.. (2022). Effects of continuous cropping on agronomic traits, root morphology, and soil nutrients in tobacco. J. Jiangsu Agric. Sci. 50 (02), 67–72. doi: 10.15889/j.issn.1002-1302.2022.02.011

[B29] Liu Z. Guo Q. Feng Z. Liu Z. Li H. Sun Y.. (2020b). Long-term organic fertilization improves the productivity of kiwifruit (*Actinidia chinensis* Planch.) through increasing rhizosphere microbial diversity and network complexity. Appl. Soil Ecol. 147, 103426. doi: 10.1016/j.apsoil.2019.103426

[B30] Ma W. F. Deng X. P. Du X. R. Dai F.X. Li J.Y. Zhao Z.X.. (2021). Effects of continuous cropping years on soil chemical properties and tobacco leaf yield and quality. J. Yunnan Agric. Univ. (Natural Science) 36 (6), 993–999.

[B31] Martin-Guay M.-O. Paquette A. Dupras J. Rivest D. (2018). The new green revolution: sustainable intensification of agriculture by intercropping. Sci. Total Environ. 615, 767–772. doi: 10.1016/j.scitotenv.2017.10.024 28992501

[B32] Nannipieri P. Giagnoni L. Renella G. Puglisi E. Ceccanti B. Masciandaro G.. (2012). Soil enzymology: classical and molecular approaches. Biol. Fert. Soils 48, 743–762. doi: 10.1007/s00374-012-0723-0

[B33] Pardon P. Reubens B. Reheul D. Mertens J. De Frenne P. Coussement T.. (2017). Trees increase soil organic carbon and nutrient availability in temperate agroforestry systems. Agr. Ecosyst. Environ. 247, 98–111. doi: 10.1016/j.agee.2017.06.018

[B34] Peng X. Yan X. Zhou H. Zhang Y. Z. Sun H. (2015). Assessing the contributions of sesquioxides and soil organic matter to aggregation in an Ultisol under long-term fertilization. Soil Till. Res. 146, 89–98. doi: 10.1016/j.still.2014.04.003

[B35] Qian X. Zang H. Xu H. Hu Y. Ren C. Guo L.. (2018). Relay strip intercropping of oat with maize, sunflower and mung bean in semi–arid regions of Northeast China: yield advantages and economic benefits. Field Crops Res. 223, 33–40. doi: 10.1016/j.fcr.2018.04.004

[B36] Ren P. Huang J. Chen X. Yu M. Hou D. (2018). Effects of relay intercropping of Chinese herbal medicine Aconitum carmichaelii debeaux and rice on soil properties and aconite yield. J. Sichuan Agric. Uni. 36 (3), 286–291. doi: 10.16036/j.issn.1000-2650.2018.03.002

[B37] Tang X. M. Meng X. Z. Jiang J. Huang Z.P. Wu H.N. Liu J.. (2020). Effects of intercropping sugarcane with peanuts on soil microecology in different soil layers. Chin. J. Oil Crop Sci. 42 (5), 713–722. doi: 10.19802/j.issn.1007-9084.2019318

[B38] Tian L. Shi W. (2014). Soil peroxidase regulates organic matter decomposition through improving the accessibility of reducing sugars and amino acids. Biol. Fertility Soils 50 (5), 785–794. doi: 10.1007/s00374-014-0903-1

[B39] Veres Z. Kotroczó S. Fekete I. Tóth J. A. Lajtha K. Townsend K. (2015). Soil extracellular enzyme activities are sensitive indicators of detrital inputs and carbon availability. Appl. Soil Ecol. 92, 18–23. doi: 10.1016/j.apsoil.2015.03.006

[B40] Wang M. M. (2016). Study on the obstacles of continuous cropping of tobacco in the Luohe tobacco-growing area. [Dissertation] (Zhengzhou: Henan Agricultural University). doi: 10.27117/d.cnki.ghenu.2016.000120

[B41] Yu Z. Liu J. Li Y. Jin J. Liu X. Wang G. (2018). Impact of land use, fertilization and seasonal variation on the abundance and diversity of nirS-type denitrifying bacterial communities in a mollisol in northeast China. Eur. J. Soil Biol. 85, 4–11. doi: 10.1016/j.ejsobi.2017.12.001

[B42] Zhang D. Li D. Li J. Y. Zeng S. J. Xu F. D. Ma W. H.. (2021). Effects of planting modes on soil enzyme activity and photosynthetic characteristics of flue-cured tobacco. Zhejiang Agric. Sci. 62 (2), 50–52. doi: 10.16178/j.issn.0528-9017.20210203

[B43] Zhang D. Y. Wang J. Yang S. P. Zhang X. Liu J. Zhao J.. (2017). Effects of Codonopsis pilosula intercropping with tobacco on soil bacterial community structure. Acta Prataculturae Sin. 26 (6), 120–130.

[B44] Zhang M. Wang N. Zhang J. Hu Y. Cai D. Guo J.. (2019). Soil physicochemical properties and the rhizosphere soil fungal community in a mulberry (Morus alba L.)/Alfalfa (Medicago sativa l.) intercropping system. Forests 10 (2), 167. doi: 10.3390/f10020167

[B45] Zhao J. Xie H. J. Zhang J. (2020). Analysis of microbial diversity and physicochemical properties in the rhizosphere microenvironment of salt-alkaline soil in the Yellow River Delta. Environ. Sci. 41 (3), 1449–1455. doi: 10.13227/j.hjkx.201908044 32608648

[B46] Zhao F. Zhao M. Z. Wang Y. Guan L. Pang F. H.. (2019). Study on the structure and diversity of strawberry rhizosphere microbial community based on high-throughput sequencing. Soils 51 (1), 51–60. doi: 10.13758/j.cnki.tr.2019.01.008

[B47] Zhou G. Yin X. Li Y. Zhao Z. Xu L. Ding J. (2015). Optimal planting timing for corn relay intercropped with flue-cured tobacco. Crop Sci. 55 (6), 2852–2862. doi: 10.2135/cropsci2014.05.0396

[B48] Zhou X. Yu G. Wu F. (2011). Effects of intercropping cucumber with onion or garlic on soil enzyme activities, microbial communities and cucumber yield. Eur. J. Soil Biol. 47 (5), 279–287. doi: 10.1016/j.ejsobi.2011.07.001

